# Evaluation of Electrolyte Concentration and Pro-Inflammatory and Oxidative Status in Dogs with Advanced Chronic Kidney Disease under Dietary Treatment

**DOI:** 10.3390/toxins12010003

**Published:** 2019-12-19

**Authors:** Doris Pereira Halfen, Douglas Segalla Caragelasco, Juliana Paschoalin de Souza Nogueira, Juliana Toloi Jeremias, Vivian Pedrinelli, Patrícia Massae Oba, Bruna Ruberti, Cristiana Fonseca Ferreira Pontieri, Marcia Mery Kogika, Marcio Antonio Brunetto

**Affiliations:** 1 School of Veterinary Medicine and Animal Science, University of São Paulo, Av. Prof. Dr. Orlando Marques de Paiva, 87, Cidade Universitária, São Paulo, SP 05508-270, Brazil; dorisph2@yahoo.com.br (D.P.H.); mv.douglas@yahoo.com.br (D.S.C.); vivian.pedrinelli@gmail.com (V.P.); brunaruberti@usp.br (B.R.); mabrunetto@usp.br (M.A.B.); 2 Animal Sciences Department, College of Agricultural, Consumer & Environmental Sciences, University of Illinois at Urbana-Champaign, Champaign, IL 217-333-3131, USA; juliana_nog@hotmail.com (J.P.d.S.N.); obapm@illinois.edu (P.M.O.); 3 Nutrition Development Center, Grand Food Industria e Comercio Ltda (Premier Pet), Dourado, SP 13590-000, Brazil; jjeremias@premierpet.com.br (J.T.J.); cristiana@premierpet.com.br (C.F.F.P.)

**Keywords:** secondary renal hyperparathyroidism, oxidative stress, inflammation, canine

## Abstract

An integrated study on the effect of renal diet on mineral metabolism, fibroblast growth factor 23 (FGF-23), total antioxidant capacity, and inflammatory markers has not been performed previously. In this study, we evaluated the effects of renal diet on mineral metabolism, oxidative stress and inflammation in dogs with stage 3 or 4 of chronic kidney disease (CKD). Body condition score (BCS), muscle condition score (MCS), serum biochemical profile, ionized calcium (i-Ca), total calcium (t-Ca), phosphorus (P), urea, creatinine, parathyroid hormone (PTH), FGF-23, interleukin 6 (IL-6), interleukin 10 (IL-10), tumor necrosis factor alpha (TNF-α) and total antioxidant capacity (TAC) were measured at baseline (T0) and after 6 months of dietary treatment (T6). Serum urea, P, t-Ca, i-Ca, PTH, FGF-23, IL-6, IL-10, TNF-α and TAC measurements did not differ between T0 and T6. Serum creatinine (SCr) was increased at T6 and serum PTH concentrations were positively correlated with serum SCr and urea. i-Ca was negatively correlated with urea and serum phosphorus was positively correlated with FGF-23. Urea and creatinine were positively correlated. The combination of renal diet and support treatment over 6 months in dogs with CKD stage 3 or 4 was effective in controlling uremia, acid–base balance, blood pressure, total antioxidant capacity, and inflammatory cytokine levels and in maintaining BCS and MCS.

## 1. Introduction

Chronic kidney disease (CKD) is considered the most common renal disease in dogs [1]. Throughout the progression of this disease, several changes occur in the organism, such as disorders of calcium and phosphorus metabolism, development of secondary renal hyperparathyroidism (SRHP), and increases in serum fibroblast growth factor 23 (FGF-23), oxidative stress and inflammation [2,3,4,5,6,7,8,9,10,11,12]. There is consensus that use of renal diets is essential for decreasing the progression rate of CKD and improving the survival of affected animals [13,14,15,16,17,18,19,20]. In addition to the restriction of protein and phosphorus, ω-3 polyunsaturated fatty acids (PUFAs) can attenuate the inflammatory process of CKD, and antioxidants may attenuate oxidative stress [7,21,22,23]. FGF-23 is a phosphatonin that is involved in the pathogenesis of mineral metabolism in CKD [24]. However, few studies in animals have been conducted to study the effects of dietary modulation of FGF-23 [25,26,27,28]. SRHP is one of the consequences of CKD, and parathyroid hormone (PTH) is considered a major uremic toxin [3,5]. Reactive oxygen species (ROS) are usually formed at a low rate in renal tissue; however, in CKD, the remaining nephrons are hyperfunctioning, increasing oxidative phosphorylation and ROS production [29]. Oxidative stress generates tissue injury and inflammation, directly contributing to the progression of CKD [11,30,31] as inflammatory cytokines increase protein catabolism and inhibit appetite, exacerbating cachexia [11,32,33]. In addition, scientific evidence indicates that patients with CKD have antioxidant and ω-3 PUFAs deficiencies and that those nutrients are capable of reducing inflammation [6,7,23,34]. Although studies have reported associations among oxidative stress, inflammation, and mineral metabolism, few have investigated dietary interactions with these parameters in dogs. The present study aimed to evaluate the effects of a renal diet in dogs with CKD stage 3 or 4, on mineral metabolism, blood pressure, acid–base balance, body condition score (BCS), muscle condition score (MCS), inflammatory cytokine levels, and total antioxidant capacity (TAC).

## 2. Results

### 2.1. Animals

Ten client-owned dogs were enrolled with a mean ± SD body weight of 16.33 ±14.64 kg, a mean age of 8.89 ± 4.46 years, various breeds and seven out of 10 were females and three of them were sterilized. Diagnosis of CKD was based on persistent azotemia over 3 months as well as imaging (ultrasound) findings of chronic kidney abnormalities. CKD classification was according to IRIS and 80% of CKD dogs were in stage 3 at T0 [35]. All animals had good acceptance of the diet, though one animal experienced an episode of diarrhea during the diet change. Three animals needed treatment with 1–2 mg/kg BW H_2_ blockers (ranitidine) every 8 or 12 h and sodium bicarbonate according to blood gas results. Two animals required 0.5–1 mg/kg BW appetite stimulants (cyproheptadine hydrochloride) every 12 h, and five animals were treated with 30–60 mg/kg BW/day phosphorus binder (aluminum hydroxide). One animal had an episode of acute decompensation of CKD and received medical treatment. Dogs were also closely monitored to detect dehydration and no parenteral fluid administration was needed, excepted for that dog that had acute renal dysfunction. Free access of tap water was assured.

### 2.2. Body Weight, BCS and MCS

A reduction in body weight was observed between T0 (16.34 ± 14.64 kg) and T6 (14.78 ± 13.32 kg; *p* = 0.045). However, BCS (T0: 5.70 ± 0.67 versus T6: 5.30 ± 1.05) and MCS (T0: 2.20 ± 0.42 versus T6: 2.40 ± 0.51) showed no significant differences (*p* = 0.375 and *p* = 0.625, respectively) between T0 and T6.

### 2.3. Urine Profile

Urinary pH remained within the reference range during the experiment, without a difference between T0 and T6 (*p* = 0.687). No differences in urine specific gravity (USG) were found between the two time points, showing isosthenuria at both times (*p* = 0.384) (Table 1). Proteinuria by semi quantitative method was not found by urine dipstick analysis along the period of observation.

### 2.4. Blood Pressure

Systolic blood pressure was within the reference range [36] and showed no difference (*p* = 0.853) between T0 and T6 (138.4 ± 5.49 mmHg) (Table 1).

### 2.5. Acid–Base Balance

Blood pH (*p* = 0.704) and blood bicarbonate concentration (*p* = 0.072) showed no differences between the two time points and were within the reference range (Table 1).

### 2.6. Biochemical Profile

Serum triglycerides (T0: 47.70 mg/dL versus T6: 41.29 mg/dL; RI, 20–112) and serum cholesterol were slightly elevated at T0 and T6 (T0: 294 ± 80.20 mg/dL versus T6: 274.40 ± 100.14 mg/dL, RI 125–270 mg/dL), but only cholesterol was significantly different between assessment periods (*p* = 0.0350). Sodium (T0: 146.36 mEq/L versus T6: 148.54 mEq/L; RI, 143–148 mEq/L), potassium (T0: 4.53 mmol/L versus T6: 4.66 mmol/L; RI, 4.37–5.65 mmol/L), alanine aminotransferase (ALT) (T0: 54.45 UI/L versus T6: 35.29 UI/L; RI, 10–88 UI/L) and alkaline phosphatase (ALP) (T0: 40.87 UI/L versus T6: 27.59 UI/L; RI, 20–150 UI/L) were within the reference ranges at both time points. Moreover, serum total protein (T0: 6.12 ± 0.45 g/dL versus T6: 6.17 ± 0.63 g/dL; RI 5.3–7.6 g/dL) and albumin (T0: 3.23 ± 0.16 g/dL versus T6: 3.18 ± 0.42 g/dL; RI 2.3–3.8 g/dL) did not differ between T0 and T6 (*p* = 0.777 and *p* = 1.000, respectively). The packed cell volume (PCV) showed a decrease between T0 (35.3 ± 6.83%) and T6 (31.2 ± 6.74%; *p* = 0.032) and was below reference values at both points (RI 37–55%). Notably, serum creatinine (SCr) was increased at T6 (*p* = 0.0022; Table 2). Serum urea, phosphorus, total calcium (t-Ca) and ionized calcium (i-Ca) showed no difference between time points (*p* = 0.187, 0.630, 0.312, and 0.232, respectively; Table 2).

### 2.7. PTH and FGF-23

Serum PTH and FGF-23 showed no difference between T0 and T6 (*p* = 0.125 and 0.858, respectively; Table 2).

### 2.8. Correlation between PTH, Total Calcium, Ionized Calcium, Phosphorus, FGF-23, Creatinine and Urea

PTH concentration was positively correlated with creatinine (*r* = 0.45; *p* < 0.05) and urea (*r* = 0.67; *p* < 0.01), and i-Ca had a negative correlation with urea (*r* = −0.59; *p* < 0.01). Urea and SCr were positively correlated (*r* = 0.45; *p* < 0.05), and FGF-23 was positively correlated with phosphorus (*r* = 0.51; *p* < 0.05).

### 2.9. Cytokines and TAC

The serum cytokines interleukin 6 (IL-6), interleukin 10 (IL-10), and tumor necrosis factor alpha (TNF-α) and TAC showed no difference between T0 and T6 (*p* = 0.148, 0.627, 0.289, and 0.6758, respectively; Table 3).

## 3. Discussion

Few studies have evaluated the role of the renal diet in CKD at different stages of the disease [14,15,20]. In the present study, the effects of a renal diet on advanced CKD in dogs were evaluated, and most of the parameters assessed were stable after feeding with renal diet and therapeutic support. Although some animals lost weight, the dietary intake was able to maintain an ideal BCS [37] and prevent the loss of MCS in the majority of animals. The maintenance of a BCS over a 3/9 score has been associated with a lower mortality rate and is an indicator of better prognosis in CKD patients [38,39].

The severity of anemia is proportional to the loss of kidney function [1], and in this study, PCV decreased between T0 and T6. Almost all dogs were able to maintain acid–base balance. Since there is an increased glomerular filtration rate (GFR) per nephron, the excretion of fixed buffers is also increased, and consequently, the excretion rate of acid titratable by the kidneys remains stable until the advanced stages of CKD [40]. Additionally, dogs with stage 3 CKD had higher bicarbonate resorption in the proximal and distal tubules compared to dogs in stage 1, showing renal adaptation, even in advanced CKD stages [41].

Feeding a renal diet did not prevent the increase in SCr; thus, the progression of renal disease was not avoided. Although the renal diet cannot itself prevent the progression of the disease, it may help to increase longevity and improve quality of life [14]. Serum phosphorus stability during the trial suggests that the combination of coadjuvant diet and therapeutic treatment was effective in maintaining a constant concentration, even with advanced CKD. Dietary restriction of phosphorus contributes to slowing the disease progression rate, as it helps to maintain phosphorus concentrations [1].

Accumulation of phosphate, caused by renal impairment, favors ionized hypocalcemia [1,42]. Both serum i-Ca and t-Ca levels remained constant during the trial, which may be positive since hypocalcemia is frequent in CKD patients, especially in the advanced stages [3,5,43]. In previous studies, the prediction of serum calcium concentration by t-Ca measurement in dogs with CKD had a 55% diagnostic discordance [42], and the correlation between t-Ca and i-Ca indicated that only 25% of the variation in t-Ca could be explained by i-Ca variation [43].

Serum PTH did not differ between T0 and T6. High levels of PTH were already expected, and one objective of this study was to maintain the stability of PTH since there is evidence that the development of SRHP occurs in the early stages of CKD and reaches up to 100% in stage 4 [3,5]. There was considerable variation in the results at T0 and T6, which may be explained by the small number of animals. Notably, high variance in PTH results was also reported in another study [44]. The main regulatory factor of PTH secretion is serum calcium, which varies inversely with its concentration, and small i-Ca oscillations cause large variations in PTH secretion [40]. In a previous study on dogs with CKD, the renal diet was unable to prevent the increase in PTH, although the animals had fewer uremic crises and increased survival time compared to the group fed a maintenance diet [15]. In the present study, serum PTH showed a positive correlation with creatinine and urea, but the correlation with phosphorus was not significant, although other studies have identified a positive correlation between PTH and phosphorus [3,5,44,45]. Similar to the present study, a previous report did not find a relationship between PTH and phosphorus and suggested that other variables in addition to phosphorus and calcium may be involved in the regulation of PTH. The positive correlation with creatinine and urea was expected because, with the deterioration of renal function, PTH concentrations tend to increase as a compensatory mechanism to maintain phosphate concentrations [4].

The increase in FGF-23 has been shown to occur prior to changes in calcium, phosphorus or PTH concentrations, which is why this metabolite has been considered one of the first biomarkers of CKD [12,25,27,46,47]. Therefore, high concentrations of FGF-23 were already expected in animals in advanced stages of the disease. Mean FGF-23 values were higher in dogs with CKD fed a renal diet than in healthy animals [26]. In the present study, the mean FGF-23 concentrations remained stable after consuming the renal diet for six months, which may have occurred because the concentrations of phosphorus, PTH, i-Ca, and t-Ca remained stable as well. In a study on cats diagnosed with CKD, feeding a renal diet for 12 months was able to reduce serum FGF-23, phosphate and PTH in hyperphosphatemic cats [28]. In the present study, a positive correlation between phosphorus and FGF-23 was found, which was expected since FGF-23 is a phosphatonin and the small number of animals could have made it difficult to find other correlations between these parameters.

The maintenance of values of TNF-α, IL-6, and IL-10 may be considered a positive result since they are usually increased in humans with CKD [48,49] due to decreased renal clearance and increased production of proinflammatory cytokines [10]. IL-10 is mainly eliminated by the kidneys, and thus, its plasma half-life may be increased in renal failure [11,50,51]. Furthermore, diets are known to influence the reduction in inflammation through the action of ω-3 PUFAs [7,21,52,53,54]. Higher ω-3 concentrations can modulate the production of eicosanoids, which is less potent in inducing vasoconstriction and aggregation [7,21,52]. Therefore, higher concentrations of ω-3 PUFAs, as used in the present study, may be required to control the immune response in advanced CKD stages.

No difference in TAC was found in the literature between CKD and healthy cats and dogs, corroborating the results found in this study and suggesting that systemic antioxidant defense systems might not be exhausted in CKD [8,55,56]. Evidence indicates that CKD is a pro-oxidative state [57,58,59,60,61] and that malnutrition status in advanced CKD patients usually culminates in a reduction in antioxidant vitamins [6,7]. This study cannot ensure that the diet was fundamental to the stabilization of TAC because all animals consumed the same diet enriched with antioxidants, but dietary supplementation with antioxidants is known to benefit CKD animals, reducing oxidative stress [22,23].

The limitations of the present study were the small size of the sample, no control group, and no urine protein:creatinine ratio (UPC) analysis, as well as different treatments that had to be initiated to control clinical and laboratory alterations. The best delineation would be the inclusion of a group of animals with CKD fed with a maintenance diet. However, the use of a maintenance diet in this situation may reduce the life expectancy of the dogs included in the control group. In advanced stages of CKD, finding animals with stable renal function and no appetite changes is difficult.

## 4. Conclusions

In conclusion, feeding dogs with advanced CKD with a renal diet for 6 months in combination with support treatment was effective in controlling uremia, acid–base balance, blood pressure, antioxidant capacity, and production of inflammatory cytokines, as well as in the maintenance of BCS and MCS. Further studies are needed to better explore how a renal diet can improve the quality of life in CKD dogs.

## 5. Materials and Methods 

### 5.1. Animals and Study Design

This study was conducted at the Veterinary Hospital of the School of Veterinary Medicine and Animal Science of the University of São Paulo (FMVZ/USP), São Paulo—SP, Brazil and approved by the Ethics Committee of the Veterinary Medicine and Animal Science School of the University of São Paulo (FMVZ/USP) on 4 September 2013, protocol number 3138/2013. The research was a prospective, 6-month longitudinal dietary trial utilizing a before (T0) and after (T6) design. Ten client-owned dogs with CKD at stage 3 or 4 [35] were included. The dogs had a stable renal function, without symptoms such as anorexia or impairment of appetite, nausea/vomiting or associated conditions, and had not consumed a renal diet previously.

### 5.2. Diet and Feeding Protocol

Nutritional contents of the diet were balanced and met all requirements for the maintenance of adult dogs [62,63]; however, the diet had a baseline concentration of protein and phosphorus and the addition of ω-3 PUFAs and vitamin E, as described in Table 4. Owners received the recommendation that no other food should be provided. Adherence to the balanced diet was assessed through a questionnaire applied monthly when the dogs returned to the hospital for recheck. There was a 4 day adaptation period to the new diet when prior food and the experimental diet were mixed (Appendix A—a). The amount of food to be fed daily was calculated using the equation: 95 kcal × (body weight) 0.75/day [62]. The result was divided by the metabolizable energy of the diet to calculate daily food intake.

### 5.3. Determination of Blood Pressure, BCS and MCS

Systolic blood pressure was measured indirectly with ultrasonic Doppler (Parks Medical^®^ Model 811-B, Oregon, United States). Blood pressure was evaluated before any clinical examination or sample collection was performed. Six measurements were performed at each time point, and the mean value was recorded. The BCS was determined according to a nine-point scale [37], and MCS was determined using a four-point scale [64]. The same trained veterinarian determined the BCS and MCS at all times to reduce possible differences in the evaluation due to subjectivity.

### 5.4. Blood, Urine Sampling and Laboratory Evaluation

The periods of blood and urine collection were labeled as times, with T0 before animals received the diet, followed by subsequent times T1 (30 days after T0), T2 (60 days after T0), T3 (90 days after T0), T4 (120 days after T0), T5 (150 days after T0) and T6 (6 months after T0). After a 12 h fasting period, blood samples were obtained from all dogs for a complete blood count (CBC) and serum biochemistry [urea, SCr, albumin, total protein, globulins, glucose, triglycerides, cholesterol, ALP, ALT, sodium, potassium, i-Ca, t-Ca, and phosphorus]. For analysis of PTH, FGF-23, cytokines and TAC, blood samples were collected and immediately placed on ice and centrifuged at 4 °C later. Serum aliquots of 1 mL were stored at −80 °C. Urine samples were obtained by cystocentesis for urinalysis (urine dipstick analysis), USG (by refractometry method), urine culture and sediment microscopic examination. Blood pH and bicarbonate measurements were performed using a blood gas analyzer (Appendix A—b); blood was collected in syringes coated with lithium heparin (Blood Gas Monovette, Sarstedt, Nümbrecht, Germany) (Appendix A—b).

### 5.5. Additional Laboratory Evaluations

For PTH, serum samples stored at −80 °C were shipped to a reference laboratory (Appendix A—c), and the concentration was determined by radioimmunoassay. Samples used to assess TNF-α, IL-6 and IL-10 cytokines were sent to a commercial laboratory (Appendix A—d) and quantified by a cytokine panel (Appendix A—e) validated for dogs; TAC samples were sent to a commercial laboratory and measured using a commercial kit (Appendix A—f). TAC was measured by quantitative colorimetric assay. In this method, Cu^2+^ is reduced to Cu^+^, and the resulting Cu^+^ forms colorful complexes. The color intensity at 570 nm is proportional to the amount of TAC in the sample, and the antioxidant concentrations were expressed in μm Trolox equivalents. FGF-23 was analyzed with a human-specific FGF-23 ELISA (Appendix A—g) validated for dogs [26] according to the manufacturer’s protocol, including dilution with the zero standard supplied in the assay kit.

### 5.6. Statistical Analysis

Statistical analyses were performed using statistical software—Statistical Analysis System (SAS) (Appendix A—h) (Statistical Analysis System 8.2, SAS Institute Inc., Cary, NC, USA). Data were assessed for normality by the Shapiro–Wilk test and tested for homogeneity by the F test. The results were arranged in 2 groups: CKD T0 (CKD dogs at baseline) and CKD T6 (CKD dogs after 6 months). The data were analyzed by a paired T-test (CKD T0 vs. CKD T6). If, after transformations, the results still had not reached normality and/or homogeneity, the data were analyzed by nonparametric statistics (SAS PROC NPAR1WAY) by the Wilcoxon test, corresponding to a paired T-test. The correlations between variables were analyzed by nonparametric statistics (Spearman correlation). Significance was set at *p* ≤ 0.05. Although some variables were assessed throughout the study (every 30 days), the values at T0 and T6 were used for the statistical analyses. The data are expressed as the mean ± SD.

## Figures and Tables

**Table 1:** Urine pH, urine specific gravity, systolic blood pressure, blood pH and blood bicarbonate concentration parameters during the 6-month follow-up compared to the initial value.

Variables	T0 (n = 10)	T6 (n = 10)	*p* Value
Urine pH	6.50 ± 1.08	6.40 ± 0.96	0.687
Urine specific gravity	1.014 ± 0.006	1.016 ± 0.004	0.384
Systolic blood pressure (mmHg)	138.8 ± 8.3	138.4 ± 5.49	0.853
Blood pH	7.33 ± 0.04	7.32 ± 0.07	0.704
Blood bicarbonate (mEq/L)	21.36 ± 1.96	19.64 ± 2.67	0.072

T0, baseline; T6, 6-month time point. Data are presented as the mean ± SD.

**Table 2:** Serum urea, creatinine, phosphorus, total calcium, ionic calcium, PTH and fibroblast growth factor 23 (FGF-23) concentrations and number of dogs who had increased or decreased parameters during the 6-month follow-up compared to the initial value.

Variables	T0 (n = 10)	T6 (n = 10)	*p* Value
Creatinine (mg/dL)	3.11 ± 1.06	4.30 ± 1.71	0.002
Urea (mg/dL)	206.07 ± 44.69	235.34 ± 74.24	0.187
Phosphorus (mg/dL)	5.15 ± 1.46	5.36 ± 1.05	0.630
Total calcium (mg/dL)	11.33 ± 1.11	11.94 ± 1.85	0.312
Ionized calcium (mmol/L)	1.41 ± 0.08	1.38 ± 0.11	0.232
PTH (pg/mL)	145.81 ± 190.69	336.48 ± 392.48	0.125
FGF-23 (pg/mL)	5645.67 ± 4720.67	5788.56 ± 5655.2	0.858

T0, baseline; T6, 6-month time point; PTH, parathyroid hormone; FGF-23, fibroblast growth factor 23. Data are presented as the mean ± SD.

**Table 3:** Serum concentrations of the IL-6, IL-10 and TNF-α cytokines and the number of animals that had increased or decreased cytokine levels at the 6-month follow-up.

Variables	T0 (n = 10)	T6 (n = 10)	*p* Value
IL-6 (pg/mL)	52.67 ± 116.28	97.26 ± 138.13	0.148
IL-10 (pg/mL)	6.93 ± 10.11	9.04 ± 11.16	0.627
TNF-α (pg/mL)	9.34 ± 20.14	15.87 ± 23.65	0.289
TAC (µmol) [3]	50.64 ± 46.74	62.71 ± 62.63	0.675

T0, baseline; T6, 6-month time point; IL-6, interleukin 6; IL-10, interleukin 10; TNF-α, tumor necrosis factor alpha; TAC, total antioxidant capacity. Data are presented as the mean ± SD.

**Table 4:** Diet composition * as fed and per 1000 kcal and ingredients according to the manufacturer.

Nutrients		
	Per 100 g of diet	Per 1000 kcal
Dry matter (g)	90.00	-
Protein (g)	14.50	35.60
Fat (g)	18.00	44.20
Ash (g)	5.50	13.50
Crude fiber (g)	3.50	8.60
Minimum calcium (g)	0.40	0.98
Maximum calcium (g)	0.90	2.21
Phosphorus (g/kg)	0.30	0.74
Potassium (g/kg)	0.60	1.47
Omega 6 (g)	2.00	4.91
Omega 3 (g)	0.52	1.27
EPA + DHA ** (g)	0.35	0.86
Food base excess (mEq)	11.30	27.75
Metabolizable energy (Kcal/g)	4.072 ***	

* Premier Nutrição Clínica Renal Cães. ** Eicosapentaenoic and Docosahexaenoic *** Metabolizable energy of the diet, previously calculated in a metabolism assay at the Premier Pet Center for Nutritional Development. * Ingredients: poultry meal, soy protein isolate, dried egg spray, broken rice, ground whole corn, barley, beet pulp, poultry fat, stabilized animal fat, fish oil, hydrolyzed poultry, antioxidants Buthylated Hydroxyanisole, potassium citrate, potassium chloride, dried brewer’s yeast, vitamin and mineral premix.

## Appendix A

^a^ The daily food requirement was calculated according to the NRC- National Research Council (NRC). *Nutrient Requirements of Dogs and Cats* (2006) adult dogs energy requirements for adult dogs, equation: 95 × (BW) 0.75 = Kcal/day divided by diet metabolizable energy;

^b^ OMNI C, COBAS B 121—Roche^®^;

^c^ Diagnostic Center for Population and Animal Health (DCPAH, Michigan State University, East Lansing, Michigan, USA);

^d^ Specialized Laboratory in Scientific Analysis—LEAC, São Paulo—SP;

^e^ Milliplex™ MAP kit CCYTO-90K-03 (MILLIPORE, Billerica, Massachusetts, USA);

^f^ Quantichrom™ Antioxidant Assay (DTAC-100, Bioassay Systems, California, USA);

^g^ Kainos, Tokyo, Japan;

^h^ Statistical Analysis System 8.2, SAS Institute Inc., Cary, NC, USA.
